# Neuropsychological Effects of Anticonvulsants

**DOI:** 10.15844/pedneurbriefs-29-7-2

**Published:** 2015-07

**Authors:** Lindsey Morgan, Alexandra Shaw

**Affiliations:** 1 Division of Neurology, Ann & Robert H. Lurie Children's Hospital of Chicago, Chicago, IL; 2 Departments of Pediatrics and Neurology, Northwestern University Feinberg School of Medicine, Chicago, IL

**Keywords:** Levetiracetam, Carbamazepine, Epilepsy, Cognitive Effects, Neuropsychological

## Abstract

Investigators from 7 centers in Korea conducted a prospective, multicenter study with patients randomized into two open-label, parallel groups and treated with either levetiracetam (LVT) or carbamazepine (CBZ) monotherapy for newly diagnosed focal epilepsy.

Investigators from 7 centers in Korea conducted a prospective, multicenter study with patients randomized into two open-label, parallel groups and treated with either levetiracetam (LVT) or carbamazepine (CBZ) monotherapy for newly diagnosed focal epilepsy. The study's primary goal was evaluation of the neuropsychological effects of levetiracetam in comparison with carbamazepine, as well as, secondarily, the medications’ respective efficacy and tolerability. Neither group experienced a worsening of general intellectual abilities. Both medications were effective in controlling seizures. There was a trend toward more frequent adverse events in CBZ patients compared to LVT. Nevertheless, the authors recommended LVT monotherapy for newly diagnosed focal epilepsy due to larger number of patients discontinuing treatment of CBZ due to adverse events, perceived poor efficacy of CBZ, and other miscellaneous reasons. [1]

COMMENTARY. In clinical practice, it is well known that children with epilepsy are at risk for cognitive, behavioral, and emotional problems. Berg et al. demonstrated that 26.4% of children have evidence of subnormal cognitive function when first diagnosed with epilepsy, with independent risk factors including: age at onset <5 years, symptomatic etiology, epileptic encephalopathy, and current AED treatment [2]. Significantly more children with seizures (11.3%) than siblings (4.6%) have behavior problems over time; however, differences diminish as seizures come under better control [3]. One third of children with epilepsy were found to have mood disorders by Caplan et al., making them 5 times more likely to do so than healthy peers [4]. The independent effect of any single AED on cognition, behavior, or emotion, however, is challenging to study for numerous reasons. Most importantly, prolonged administration of AEDs cannot be tested on normal subjects; AEDs can theoretically produce different cognitive effects in different epilepsy syndromes and in children at different ages; recent seizures as well as drug dosages and levels modulate cognitive performance [5]. Selecting an appropriately precise series of targets for cognitive testing on can be cumbersome. While IQ provides an interesting global measure, it obfuscates the study of specific cognitive processes, such as processing speed or attention [5]. Lastly, it is difficult to construct a study sufficiently powered to show unambiguous negative or positive effects of an AED. The current study falls just below its own threshold of 42 subjects per group to provide 80% power with a 5%, 2- tailed level of significance. The cognitive side effects of many AEDs have been tested previously, with phenobarbital and topiramate well known to have adverse effects. In a prior study of adult epilepsy patients, CBZ and LVT were compared using 11 neuropsychological tests evaluating attention and memory. Across all variables tested, the effects of CBZ were worse than LVT. Both performed worse than non-drug patients: in 76% of variables for CBZ and 12% of variables for LVT [6]. These results are comparable to the current study in a pediatric population [1]. Overall, a multiplicity of interrelated factors contributes to cognitive, behavioral, and emotional problems in children with epilepsy, but seizure control and proper medication choice can mitigate these risks.

## References

[1] Jung E, Yu R, Yoon JR, Eun BL, Kwon SH, Lee YJ (2015). Neuropsychological effects of levetiracetam and carbamazepine in children with focal epilepsy. Neurology.

[2] Berg AT, Langfitt JT, Testa FM, Levy SR, DiMario F, Westerveld M (2008). Global cognitive function in children with epilepsy: a community-based study. Epilepsia.

[3] Austin JK, Perkins SM, Johnson CS, Fastenau PS, Byars AW, deGrauw TJ, Bunn DW (2011). Behavior problems in children at time of first recognized seizure and changes over the following 3 years. Epil & Behav.

[4] Caplan R, Siddarth P, Gurbani S, Hanson R, Sankar R, Shields WD (2005). Depression and anxiety disorders in pediatric epilepsy. Epilepsia.

[5] Lagae L (2006). Cognitive side effects of anti-epileptic drugs. The relevance in childhood epilepsy. Seizure.

[6] Meador KJ, Gevins A, Loring DW, McEvoy LK, Ray PG, Smith ME (2007). Neuropsychological and neurophysiologic effects of carbamazepine and levetiracetam. Neurology.

